# Computational simulations of identified marine-derived natural bioactive compounds as potential inhibitors of oral cancer

**DOI:** 10.2144/fsoa-2021-0148

**Published:** 2022-01-24

**Authors:** Prabhu Manickam Natarajan, Vidhya Rekha Umapathy, Anita Murali, Bhuminathan Swamikannu

**Affiliations:** 1 Assistant Professor in Periodontics, College of Dentistry, Ajman University, Ajman, UAE.; 2 Reader, Department of Public Health Dentistry, Sree Balaji Dental College & Hospital, Chennai, 600100. India; 3 Professor, Department of Public Health Dentistry, Sree Balaji Dental College & Hospital, Chennai, 600100, India; 4 Professor, Department of Prosthodontics, Sree Balaji Dental College & Hospital, Chennai, 600100, India

**Keywords:** ADME, Akt1, Akt2, bioactive compounds, lipinski rule of five, marine algae, molecular docking, oral squamous cell carcinoma, PyRx, virtual screening

## Abstract

Oral squamous cell carcinoma is characterized by the upregulation of RAC-alpha serine/threonine-protein kinase (Akt1) and RAC-beta serine/threonine-protein kinase (Akt2). In this work, Akt1 and Akt2 were inhibited using a cocktail of 20 marine algae chemicals. From the PyRx Virtual Screening Tool, dieckol, 6,6′-bieckol, siphonaxanthin and sargachromanol E were chosen as the best four compounds for Akt1 based on the scoring. Similarly, dieckol, 6,6′-bieckol, dioxinodehydroeckol and caulerpenyne were chosen as Akt2 inhibitors. Additionally, the results of the Lipinski rule of five indicated that some of the selected compounds, such as dieckol, 6,6′-bieckol and siphonaxanthin, violated some Lipinski rules, but they demonstrated excellent binding in terms of scoring. Thus, this study demonstrates that the identified lead compounds may act against Akt1 and Akt2 in oral cancer.

## Lay abstract

High-level expression of RAC-alpha serine/threonine-protein kinase (Akt1) and RAC-beta serine/threonine-protein kinase (Akt2) is responsible for oral cancer. Control of the expression of these target proteins will be used to control oral cancer. Marine algae are one of the best sources of anticancer compounds. Hence, 20 marine compounds were selected and their activities against Akt1 and Akt2 proteins were checked by using virtual screening techniques. The results of this screening rank the 20 compounds based on the binding score. The best four compounds for each target protein (Akt1: dieckol, 6,6′-bieckol, siphonaxanthin and sargachromanol E; Akt2: dieckol, 6,6′-bieckol, dioxinodehydroeckol and caulerpenyne) were selected. Expect for a few compounds most of the compounds satisfied the drug likeness quality. So dieckol,6,6'-bieckol,dioxinodehydroeckol and caulerpenyne, siphonaxanthin and sargachromanol E. may be a potential lead to further drug development for oral cancer.

## Graphical abstract

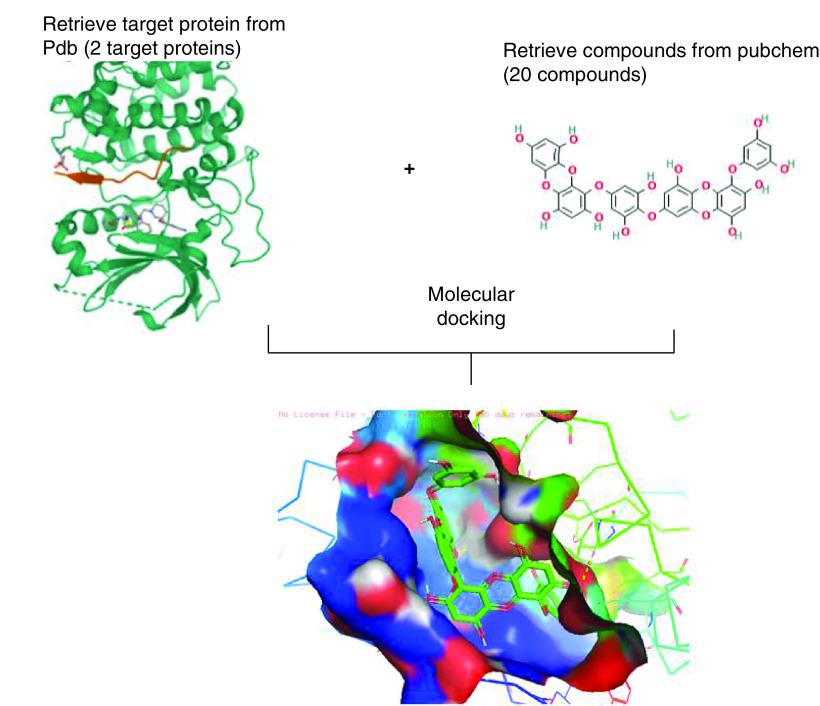

Oral cancer is among the most difficult diseases that humanity faces, and despite numerous advances in the field of oral cancer detection and treatment, it continues to be a major public health problem worldwide. Oral malignancies are predominantly carcinomas (96%), with squamous cell carcinomas accounting for 91% of all cases. Differences in the prevalence of this cancer are caused by a variety of endogenous and exogenous variables, including cigarette usage, alcohol consumption and infection with the papilloma virus (PV). Numerous genetic and epigenetic alterations are induced as a result of these variables, resulting in genomic destabilization and tumor growth and development [1–6]. Despite a high mortality rate and a reduced heal percentage, the total and disease-free survival rates of patients with oral squamous cell carcinoma (OSCC) stay stable. This is mostly due to a dearth of appropriate preventive and surgical biological markers for improved prediction and diagnosis, as well as a dearth of effective treatments [7,8]. As a result, it has become important to concentrate on the molecular mediators implicated in the genesis and development of oral cancer.

Many decades of study have proven that the protein kinase B (Akt)/mammalian target of rapamycin (mTOR) pathway is substantially elevated in oral cancer. The stated threats for oral cancer such as cigarettes, alcohol and human PV have also been reported to cause initiation of the Akt/mTOR pathway [9,10]. Such a path is a network of several receptors that communicate and drive diverse biological activities such as cancer cell survival, multiplication, incursion, angiogenic activity and tumor spread. Akt kinase is the main source of protein of such a pathway and its initiation is able to promote carcinogenesis by altering a few key characteristics of cancer [11,12].

Much evidence suggests that Akt isoforms play a part in the development of various types of cancer, including ovarian, colorectal, pancreatic, breast and lung cancer [13,14]. Moreover, it is well established that Akt kinase exists in three separate isoforms known as Akt1, Akt2 and Akt3, each of which has unique activity in numerous malignancies [15]. Further, the precise involvement of Akt isoforms in oral cancer development has still not been adequately investigated. As a result, the current study sought to determine the function of various Akt isoforms in the etiology of oral cancer. Decoding the molecular network of Akt isoforms involved in the development of OSCC may provide this disease a distinct target for the development of effective prevention and therapeutic methods.

In malignancies, increased AKT activity caused by somatic mutations impairs AKT’ downstream elements. When AKT is activated, proapoptotic proteins such as Bcl-2-associated death promoter (Bad) and Bcl-2-associated X (Bax) are inhibited. Inhibiting Bad reduces its ability to regulate the anti-apoptotic B-cell lymphoma extra-large (Bcl-xL) protein, hence inhibiting the apoptotic process. AKT also inhibits caspases directly implicated in apoptosis and FOXO-1, a transcription factor that regulates the production of proapoptotic genes such as Bcl-2-like protein 11 or Bim and Fas-ligand (FasL) [16,17]. AKT has also been shown to inhibit glycogen synthase kinase 3 (GSK-3) and FOXO activity, which results in the overexpression of cyclin D1 [18,19]. When cyclin D1 is activated, it results in an increase in the expression of cyclin-dependent kinase (CDK) 4 and 6, which allows cells to exit the G1 phase and enter the replication phase, that is, the S-phase, thereby increasing proliferation. Additionally, AKT modulates the cytoplasmic location of p27, which is critical for tumor aggressiveness and metastasis [20].

As the stimulation of Akt has been an important predictive factor in OSCC, blocking it may provide a viable molecular strategy for therapies [21]. Akt or protein kinase B (PKB) is composed of three closely related isoforms: Akt1, Akt2 and Akt3 (or PKBa/b/c, separately). Three Akt isoforms are structurally and functionally identical and have comparable activation methods, but their tissue expression sites are unique [22]. Akt1 and Akt2 are highly expressed in tissue, whereas Akt3 expression is restricted. The study focuses on Akt1 and Akt2 as potential targets for OSCC.

The marine environment has indeed been demonstrated to be a valuable source of compounds with unusual and distinct chemical properties that can be used to generate novel treatments with increased potency and specificity.

Oceans constitute around 70% of the earth's surface and provide habitats for a wide variety of creatures [23]. Numerous metabolic products are produced by these species. Lower organisms, in particular, produce a plethora of secondary metabolites that serve as “defense and attack” signaling molecules. These molecules, which come from a variety of chemical classes, have the potential to be used as healthcare therapies [24]. Bacteria, fungi, corals, micro- and macroalgae, gorgonians, sponges, nudibranchs, bryozoans, sea cucumbers, tunicates and sea hares, among other marine organisms, have yielded several promising therapies in recent decades [25]. Efforts have been made to isolate these chemicals, with more than 10,000 natural products (NPs) of potential biotechnological interest identified to date. Algae are one of the ocean's most valuable commodities, both commercially and ecologically [26]. The 20 marine-based compounds have been reported to have potential anticancer activities in other types of cancers; however, only very few studies have been reported for oral cancer. Hence, based on the literature evidence, in the present study, the authors aimed to check the possible role of the 20 compounds against the molecules involved in the progression of oral cancer toward identification of potential inhibitors for oral cancer.

## Materials & methods

### Compound preparation

This study focused on 20 chemicals isolated from marine algae Table 1. The structures of the molecules were retrieved from the PubChem database in sdf format (https://pubchem.ncbi.nlm.nih.gov/). The chemicals were loaded into OpenBabel and subjected to energy minimization using the Python Prescription Virtual Screening Tool (PyRx)16. The conjugate gradient approach was used to minimize the energy in the Universal Force Field (UFF). The total number of steps was set to 200, while the number of updates was set to 1. Additionally, the reduction procedure was configured to terminate at an energy difference of less than 0.1 kcal/mol [27].

**Table 1.** List of selected compounds from marine algae.

Serial no.	Compound name
1	6,6′-Bieckol
2	Bromophycolide A
3	caulerpenyne
4	Caulerpin
5	Clerosterol
6	Dieckol
7	Dioxinodehydroeckol
8	Elatol
9	Fucodiphloroethol G
10	Fucoidan
11	Fucoxanthinol
12	Laminaran
13	Laminaran
14	Laurenditerpenol
15	Lophocladine B
16	Mertensene
17	Porphyran
18	Sargachromanol E
19	Siphonaxanthin
20	Thyrsiferol

### Protein preparation

The crystal structures of Akt1 (PDB ID: 3QKL) and Akt2 (PDB ID: -2JDO) were obtained from the Protein Data Bank ( https://www.rcsb.org/). Separation of the ligand from the protein was accomplished. The protein was supplied with polar hydrogen atoms and Kollman charges, while the water molecules were removed. The final generated file was reduced and saved in PDBQT format for further examination using the UCSF Chimera program [28].

### Structure-based virtual screening

To identify a new, powerful lead drug for Akt1,2, structure-based virtual screening (SBVS) was performed on produced compounds utilizing docking simulations. The binding energies were calculated using PyRx AutoDock VINA. To begin, a grid box covering the active region of the crystal structure was created with an exhaustiveness of 8 and set to output just the lowest energy pose. Finally, the data were analyzed using the Discovery Studio [29] and PyMOL [30] tools.

### ADME prediction

The Lipinski filter (http://www.scfbio-iitd.res.in/software/drugdesign/lipinski.jsp) has been used to determine the absorption, distribution, metabolism and excretion (ADME) of chosen natural compounds. An orally active medication must satisfy at least four of the five criteria for drug similarity, namely. molecular mass, cLogP, hydrogen donor and acceptor and molar refractive index [31].

## Results & discussion

The sixth most common cancer type in the world is head and neck cancer, which includes OSCC. In south-central Asia and Central and Eastern Europe, OSCC is among the leading causes of illness and mortality. OSCC usually affects the tongue, oropharynx, lip, floor of mouth, gingiva, hard palate and buccal mucosa, in decreasing order [32].

Members of the aldo-keto reductase (AKR) superfamily reduce the toxic and carcinogenic impacts of multiple genotoxic or non-genotoxic organic compounds and protect mammalian cells, but AKR1C2 and AKR1C3 from this family induce cancer progression by meditating phosphogluconate dehydrogenase (PGD) conversion through the phosphatidylinositol 3-kinase (PI3K)/Akt signal pathways [33]. PI3K is required for Akt isoform activation. Signaling molecules in the phosphoinositide 3-kinase (PI3K)/Akt pathway have drawn the attention of numerous researchers as therapeutic targets for cancer treatment due to their critical role in the carcinogenesis of several malignancies, including OSCC [34]. All hallmarks of cancer and malignant aggressiveness, including anti-apoptosis, cell proliferation, angiogenesis, immortalization, tissue invasion and metastasis, are associated with PI3K/Akt pathway dysregulation [35].

Following a thorough examination of Akt1, Akt2 and OSCC, the goal of this research was to identify several potential lead compounds for OSCC. Twenty ligands from marine sources were evaluated for this study. This screening was conducted using molecular docking and other *in silico* techniques. In structure-based small molecule docking, a small drug molecule is inserted into the target protein's binding cavity and the resulting docking position is evaluated using a specific scoring scheme (binding affinity) [36]. Binding affinity is usually a top priority when choosing the best candidate for virtual screening. The lowest energy value reflects the strongest ligand protein binding, as evidenced by the best docking scores [37].

It is currently the greatest way to forecast docking conformations of compounds that are energetically favorable to bind with a pharmacological receptor sites quickly, and it has grown in prominence as a way to save time and money in the drug discovery and development pipeline. Thousands of compounds can be evaluated for possible pharmacological activity using docking studies at minimal cost and in a short period of time. In this study, the authors used the docking technique to uncover novel Akt1 and Akt2 inhibitors utilizing SBVS on marine-based compounds.

Protein, fiber, vitamins, polyunsaturated fatty acids, macroelements and trace elements are all found in abundance in algae. Lately, they have been discovered to be a good source of antioxidants, phycocolloids, proteins, vitamins, minerals, carotenoids, soluble dietary fiber, polyunsaturated fatty acids, phycobilins, polysaccharides, sterols, tocopherols, terpenes and phycocyanins, among other bioactive components. Apart from their potential application as therapeutic agents in the biomedical field, these compounds have been shown to have nutritional and functional benefits [38]. Algae's multifunctional properties should be fully explored due to their unique structures and biological characteristics. Furthermore, the fact that more than 30,000 algal species have been identified around the world supports the assumption that algae are a very prolific source of structurally distinct NPs with medicinal potential. Furthermore, after sponges, bacteria and phytoplankton, algae have been identified as a key source of novel marine chemicals. Twenty compounds from marine algae were chosen for virtual screening against the OSCC target proteins Akt1 and Akt2 in this investigation. The compounds were graded based on their binding energy after the docking investigations. The best four compounds for Akt1 were picked from this output based on the score parameters: dieckol, 6,6′-bieckol, siphonaxanthin and sargachromanol E. Akt2 inhibitors were chosen from dieckol, 6,6′-bieckol, dioxinodehydroeckol and caulerpenyne.

**Figure 1.**
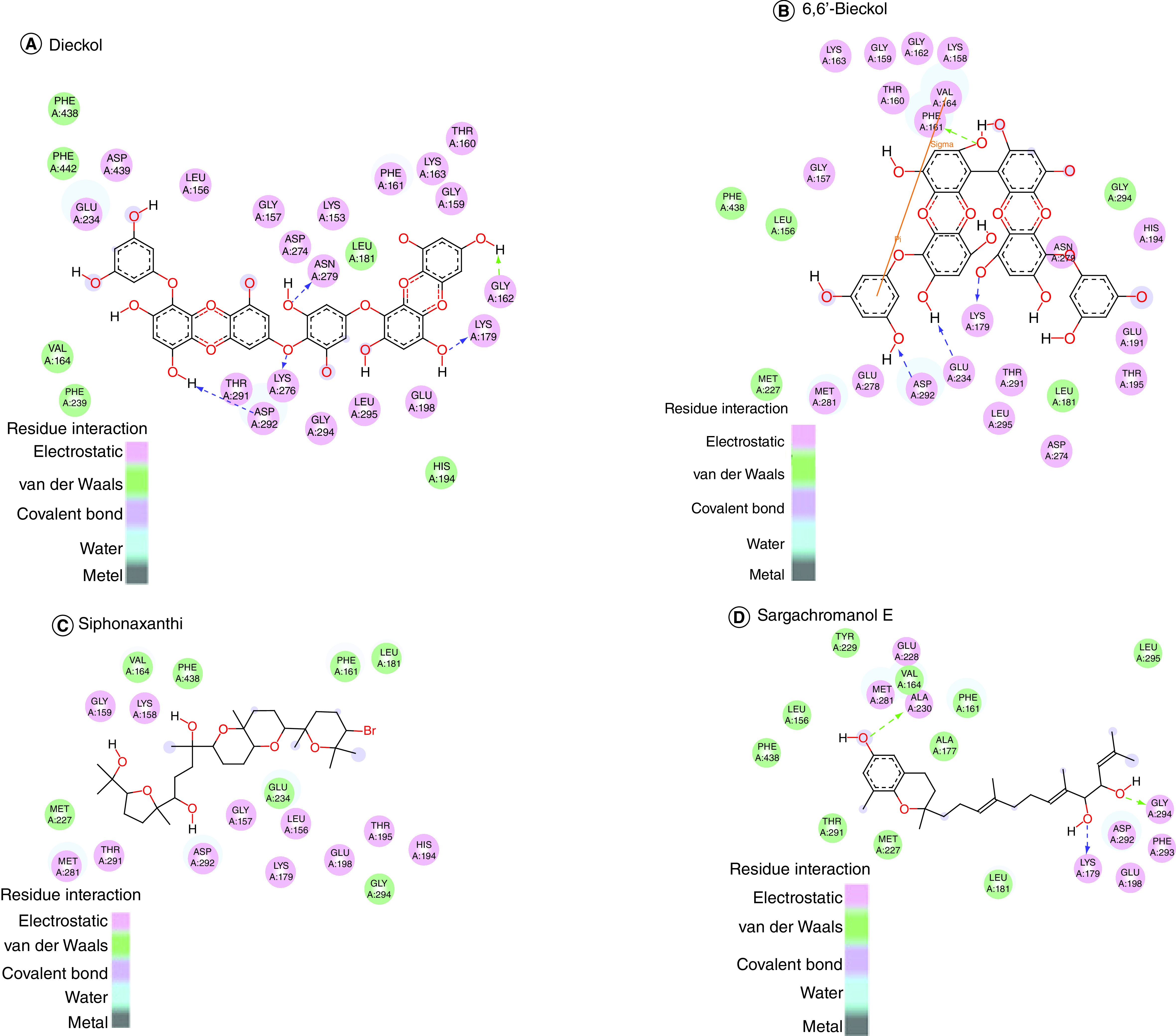
Molecular interaction of Akt1. Molecular interaction of Akt1 with: **(A)** Dieckol **(B)** 6,6′-bieckol, **(C)** siphonaxanthin and **(D)** sargachromanol E.

**Figure 2.**
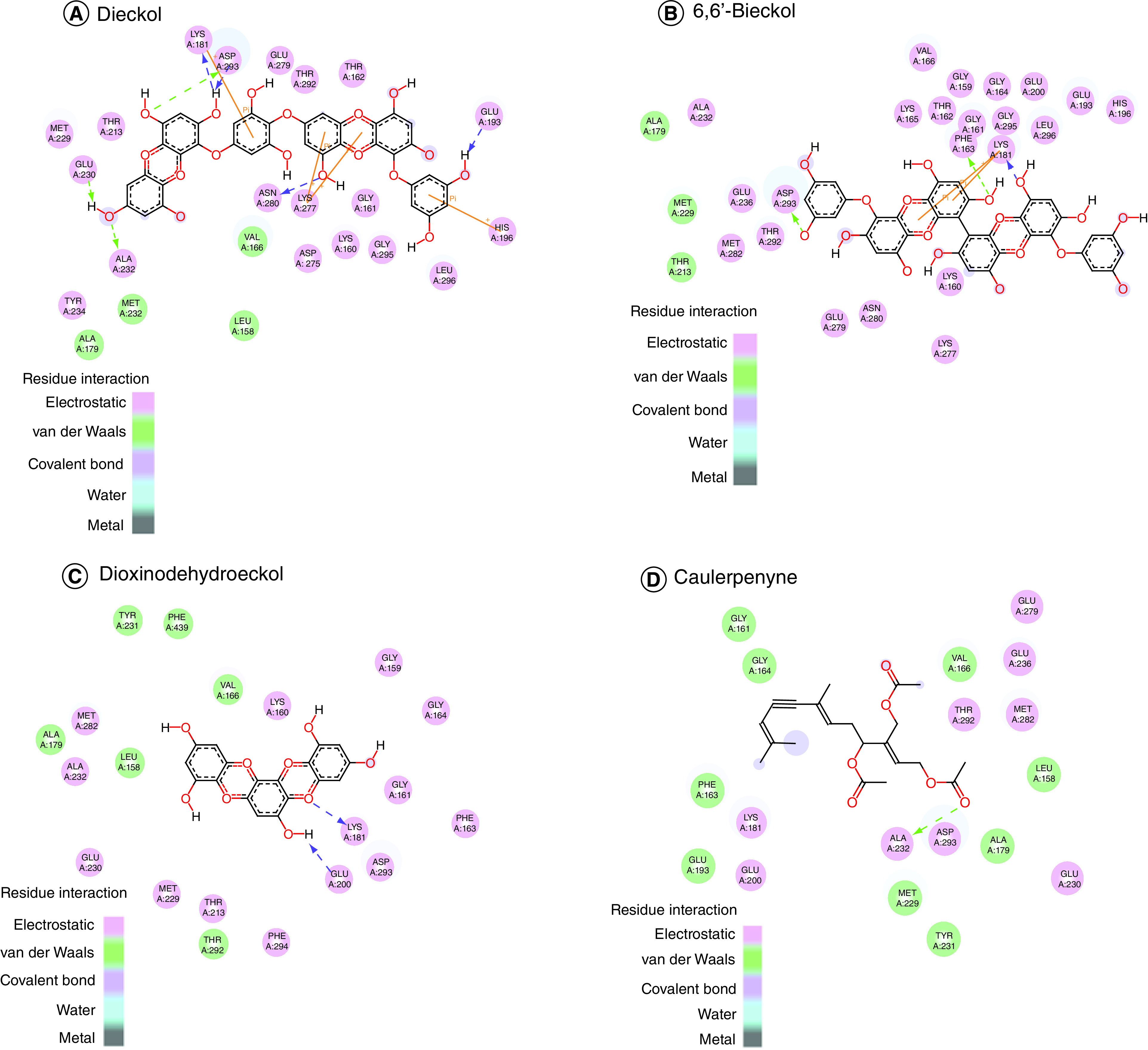
Molecular interaction of Akt2. Molecular interaction of Akt2 with **(A)** dieckol, **(B)** 6,6′-bieckol, **(C)** dioxinodehydroeckol and **(D)** caulerpenyne.

**Figure 3.**
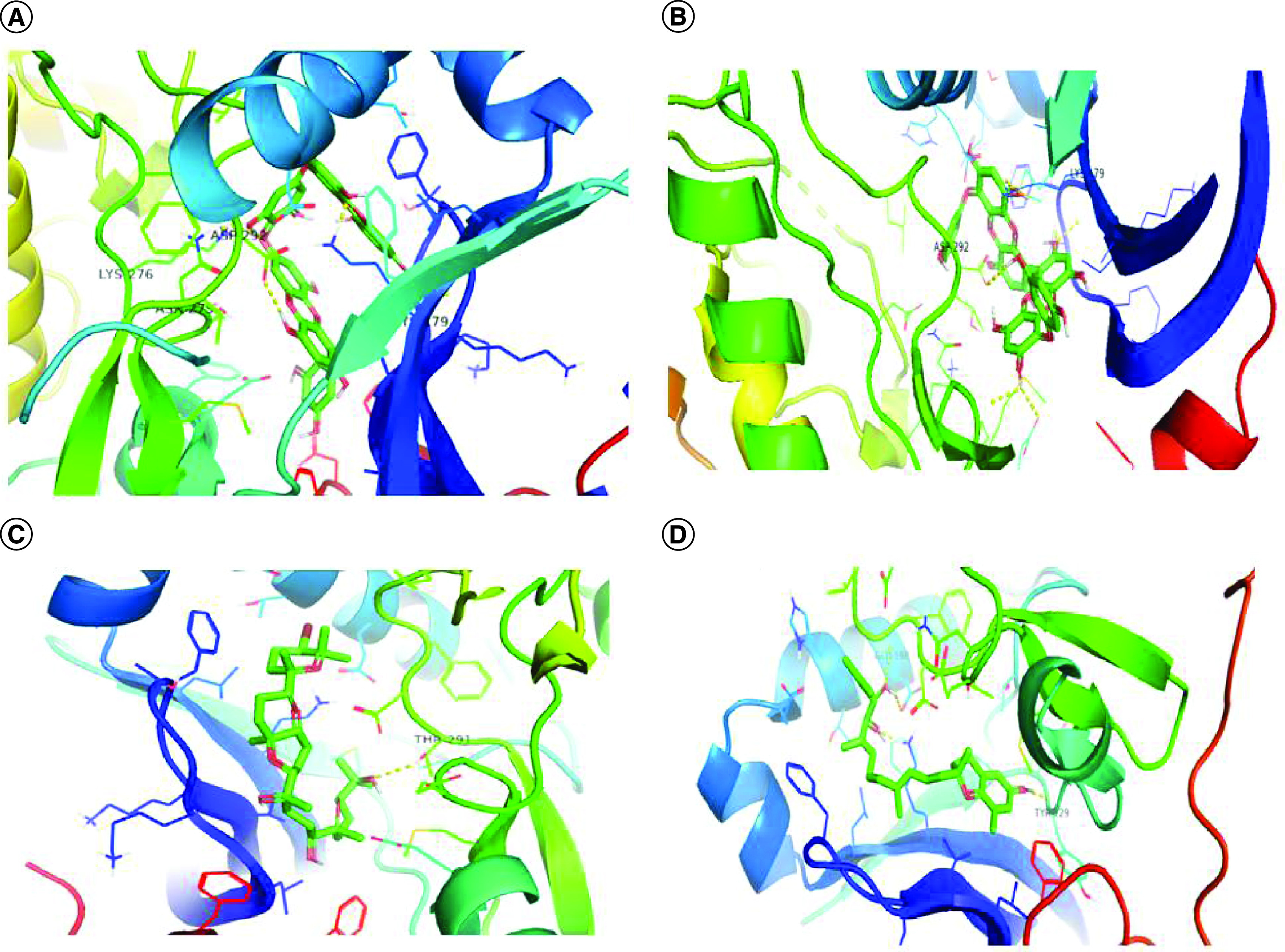
3D interaction picture of Akt1. 3D interaction picture of Akt1 with **(A)** dieckol, **(B)** 6,6′-bieckol, **(C)** siphonaxanthin and **(D)** sargachromanol E.

**Figure 4.**
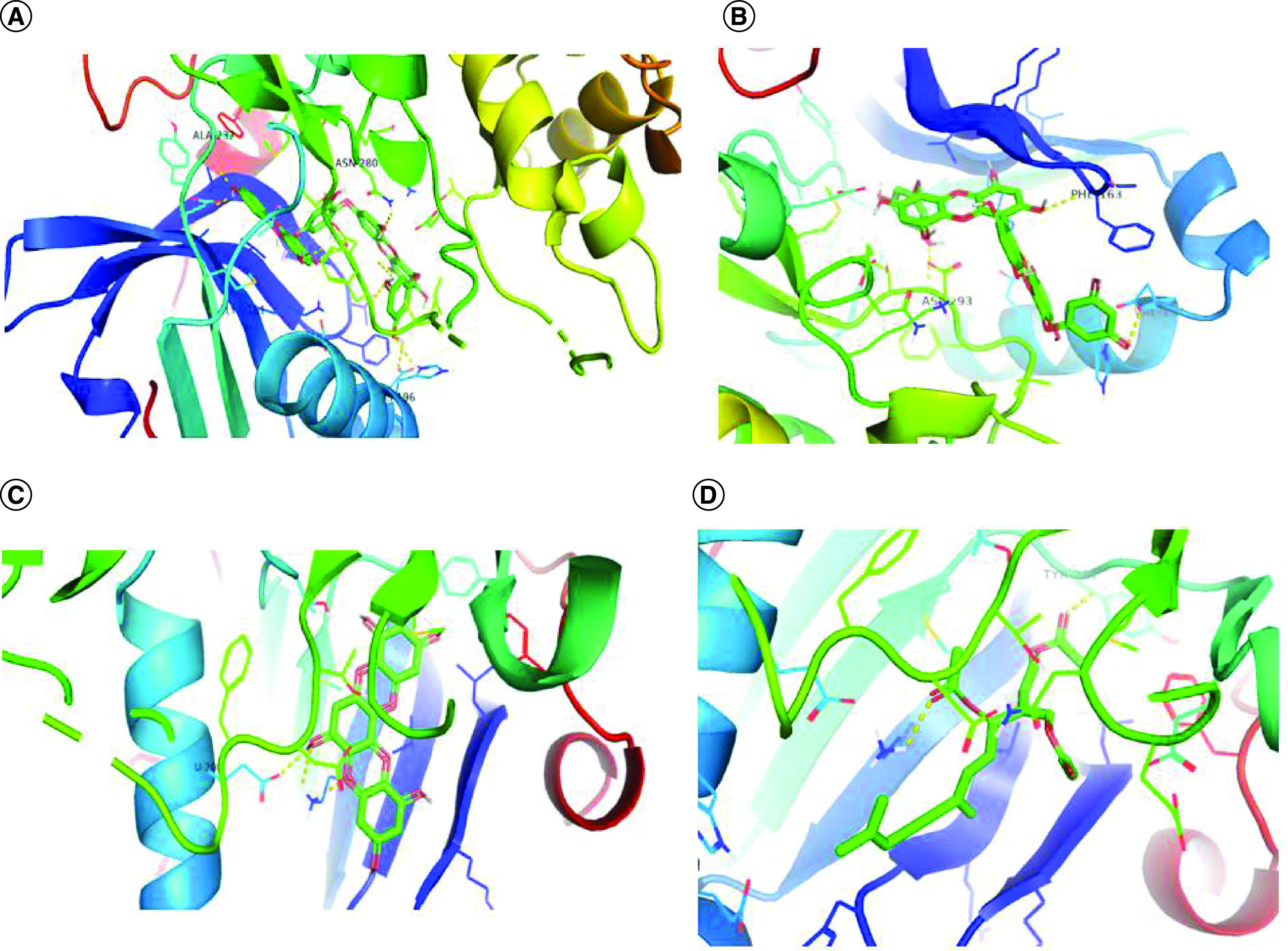
3D interaction picture of Akt2. 3D interaction picture of Akt2 with **(A)** dieckol, **(B)** 6,6′-bieckol, **(C)** dioxinodehydroeckol and **(D)** caulerpenyne.

The binding affinities of selected compounds with Akt1 (PDB ID-3QKL) were found to be in the range of -9.5 kcal/mol to -10.8 kcal/mol, indicating a significant binding affinity. The binding affinities of Akt2 (PDB ID-2JDO) and marine-based chemicals were again found to be in the range of -9.2 kcal/mol to -11.4 kcal/mol, indicating a significant binding affinity. The Visualizer in Discovery Studio was used to visualize the the protein–ligand interactions of all drugs with Akt1 and Akt2, respectively. Bonds formed among these amino acids of Akt1–ligand complexes as well as Akt2–ligand complexes are shown in Figures 1 & 2. 3D interaction of selected compounds with Akt1 and Akt2 is shown in Figures 3 & 4.

The dieckol–Akt1 complex had the strongest bonding of the four complexes, with a binding energy of -10.8 kcal/mol. It forms electrostatic and hydrogen bonds, which indicate a positive ligand–protein interaction, since these bonds are essential for forming a strong binding. In the realm of molecular chemistry, Pi–cation interactions play a crucial role [39–41]. In molecular recognition as well as chemical and biological catalysis, the pi–cation bond is crucial. Akt1–dieckol Table 2 lists the amino acids implicated between the Akt-1-Dieckol interactions in detail. Dieckol, like Akt1, demonstrated a stronger affinity for Akt2 than the other chemicals, with a binding energy of -11.4. The docked complexes were seen in PyMOL, and all of the identified compounds attached to the same binding site of Akt1 and Akt2.

**Table 2.** Results of selected compounds from docking studies.

Serial no.	Compound name	Docking score kcal/mol	Hydrogen bond interaction	Other interaction
Akt1				
1	Dieckol	‐10.8	GLY-162	ASN-279 ASP-292
2	6,6′-Bieckol	‐10.7	PHE-161	VAL-164 LYS-179 GLU-234 ASP-292
3	Siphonaxanthin	‐10.1		
4	Sargachromanol E	‐9.5	ALA-230 GLY-294	LYS-179
Akt2				
1	Dieckol	‐11.4	ASP-293 GLU-230 ALA-232	LYS-181 LYS-277 ASN-280 GLU-193
2	6,6′-Bieckol	‐10.3	ASP-293 PHE-163	LYS-181
3	Dioxinodehydroeckol	‐9.5	–	LYS-181 GLU-200
4	Caulerpenyne	‐9.2	ALA-232	–

The projected ADMET characteristics were calculated to aid future lead optimization. Except for a few compounds, the Lipinski rule is followed by the majority of the compounds. Table 3 shows the ADME properties that were predicted.

**Table 3.** Predicted absorption, distribution, metabolism and excretion properties of selected compounds.

Compound name	Mass^†^	Hydrogen bond donor^‡^	Hydrogen bond acceptors^§^	logP^¶^	Molar refractivity^#^
Dieckol	742	11	8	7.934302	181.018982
6,6′-Bieckol	742	12	18	7.201403	178.067810
Siphonaxanthin	604	7	3	5.183600	150.599487
Sargachromanol E	428	3	4	5.925322	127.624352
Dioxinodehydroeckol	379	5	9	4.323801	91.481979
Caulerpenyne	374	0	6	3.747899	101.889969

† Molecular mass less than 500 Da.

‡ Less than 5 hydrogen bond donors.

§ Less than 10 hydrogen bond acceptors.

¶ logP less than 5.

# Molar refractivity should be between 40 and 130.

## Conclusion

The major goal of this pilot study was to see if SBVS could be used to find novel inhibitors with action against Akt1 and Akt2 in oral cancer. The SBVS was carried out with the help of AutoDock VINA, which is included in the PyRx 0.8 package. The findings also point to new study directions that could be explored in future initiatives. The study was done in silico with a variety of effective ways. The outcomes of these in silico approaches generate a precise and clear sense of the efficiency of known anti-tumor medicine by interacting with the Akt pathway and providing therapeutic efficacy.By interacting with the Akt pathway and giving therapeutic efficacy, the results of these *in silico* methodologies generate a precise and clear notion of the efficiency of the established antitumor medicine. The findings of this study pointed to a promising future for this medicine as a potent alternative to the already-proven Akt inhibitors in the treatment of OSCC. As a result, dieckol, 6,6′-bieckol, dioxinodehydroeckol, caulerpenyne, siphonaxanthin and sargachromanol E in the Akt pathway can be considered for therapy of OSCC.

**Summary points**

- Oral cancer is among the most difficult diseases that humanity faces, and despite numerous advances in the field of oral cancer detection and treatment, it continues to be a major public health problem worldwide.
- The upregulation of RAC-alpha serine/threonine-protein kinase (Akt1) and RAC-beta serine/threonine-protein kinase (Akt2) is the main cause of oral squamous cell carcinoma.
- The marine environment represents a unique source of bioactive compounds with high pharmaceutical potential.
- Algae are one of the ocean's most valuable commodities, both commercially and ecologically.
- Twenty compounds from marine algae were selected for screening against Akt1 and Akt2.
- The best 4 compounds for Akt1 were picked from this screening based on the score parameters: dieckol, 6,6′-bieckol, siphonaxanthin and sargachromanol E. Akt2 inhibitors were dieckol, 6,6′-bieckol, dioxinodehydroeckol and caulerpenyne.
- The predicted absorption, distribution, metabolism and excretion showed that except for a few compounds, the Lipinski rule is followed by the majority of the compounds.
- Hence, these compounds may act as potential leads for identifying a new oral cancer drug.
